# Clinical efficacy of different shoulder joint drug injections for rotator cuff injuries: A network meta-analysis

**DOI:** 10.1097/MD.0000000000030659

**Published:** 2022-09-30

**Authors:** Fang Zhi, Feiyan Cai, Wei Zhang, Liming Xiong, Jinglin Hu, XingZhen Lin

**Affiliations:** a Nanchang Hongdu Hospital of Traditional Chinese Medicine, Nanchang, Jiangxi Province, China; b Suining First People’s Hospital in China, Suining, China.

**Keywords:** corticosteroid, hyaluronic acid, platelet-rich plasma, prolotherapy, rotator cuff injury

## Abstract

Rotator cuff injury is a common clinical disorder of the musculoskeletal system. It is one of the most common causes of shoulder pain and shoulder joint dysfunction. Intra-articular injection therapy is one of the common conservative treatments for rotator cuff injury in clinical practice, but scholars have been controversial about the different drugs used for injection therapy and their clinical effectiveness, and there is no complete agreement on the choice of different injectables, and the existing original studies are mostly direct comparisons in 1 arm, lacking indirect comparisons, making it difficult to have a clear and comprehensive understanding.

**Objective:**

A network Meta-analysis was used to comprehensively compare the effectiveness of drug injection therapies for rotator cuff injuries.

**Methods:**

The PubMed, The Cochrane Library, EMbase, Chinese national knowledge infrastructure, Chinese BioMedical Literature on disc, China Science and Technology Journal Database, and Wan-Fang databases were searched for randomized controlled trials on the effectiveness of steroid injections for the comparative treatment of rotator cuff injury for the period April 19, 2013 to April 19, 2022 (the last decade). Two investigators independently screened the literature, extracted data according to inclusion and exclusion criteria, and evaluated the quality of the literature in parallel. Statistical analysis was performed using Stata 16.0 software to compare the differences in efficacy of each treatment measure and rank the efficacy using the ratio and 95% confidence interval (CI) as the effect indicator.

**Results:**

10 RCTs with a total of 861 patients with rotator cuff injury were included, involving 4 therapeutic measures: corticosteroid injection therapy (COR), platelet-rich plasma injection therapy (PRP), Hyaluronic acid injection therapy (HA), and prolotherapy therapy (PRO).Meta-analysis results showed that the ranking results of the 4 therapeutic measures were: corticosteroid injection + hyaluronic acid injection > platelet-rich plasma injection + corticosteroid injection > corticosteroid injection > platelet-rich plasma injection > PRO > platelet-rich plasma injection + hyaluronic acid injection > hyaluronic acid injection.

**Conclusion:**

we recommend that corticosteroid injections combined with hyaluronic acid injections can be used for the non-surgical conservative clinical management of rotator cuff injuries.

## 1. Introduction

Rotator cuff injury is a musculoskeletal system disorder that causes supervision and pain and impaired movement of the shoulder joint, which seriously affects patients’ activities of daily living.^[1–3]^ Some studies have statistically found that the incidence of rotator cuff injury in people over 50 years of age is about 25%,^[4]^ and there are 200,000 to 300,000 new cases of rotator cuff injury worldwide each year.^[5]^ The increasing number of people seeking conservative treatment for this problem has also placed greater demands on the associated clinical consultation.

Several studies have found that shoulder joint drug injection therapy is one of the commonly used conservative treatments for rotator cuff injury,^[5,6]^ which has the effect of reducing shoulder pain and increasing joint mobility, thus improving the ability to perform daily life and improving the quality of survival of patients. The main methods commonly used for shoulder injections are corticosteroid injection therapy (COR), platelet-rich plasma injection therapy (PRP), Hyaluronic acid injection therapy (HA), and prolotherapy therapy (PRO), but there have been controversies about the different drugs and their clinical effectiveness, and there is no complete agreement on the choice of different injections. The existing original studies are mostly comparisons between single drug injections and lack direct comparisons between classes of multiple drug injections. Although there are several meta-analyses regarding COR, PRP, HA, and PRO for rotator cuff injuries, most of them are single therapy comparisons. Traditional meta-analysis also enables only two-to-one comparisons, not multiple treatment measures,^[7]^ which prevents researchers from comprehensively and systematically evaluating the efficacy of steroid shoulder injection modalities and injection sites, and is not conducive to the selection and promotion of optimal treatment protocols. This study focused on 4 different shoulder injection modalities: steroid injection, platelet-rich plasma injection, sodium glutamate injection, and hyperglycemic augmentation therapy, and performed a reticulated META analysis to provide a theoretical basis for selecting the optimal drug injection modality to intervene in rotator cuff injury.

## 2. Data and Methods

The article is reported in accordance with The National Institute for Health and Care Excellence (NICE) Reticulated Meta-analysis Reporting Specification.^[8]^

### 2.1. Inclusion criteria

#### 2.1.1. Study type.

A randomized clinical trial (RCT) of the comparative effectiveness of different shoulder drug injections.

#### 2.1.2. Inclusion criteria.

Patients with rotator cuff injury: pain in the anterior triangle of the shoulder joint and limitation of shoulder joint movement, confirmed by MRI; age ≥ 18 years, gender not limited; duration of disease ≤ 3 months; unilateral onset.

### 2.2. Interventions

Include at least 2 therapies from shoulder COR, PRP, HA, and PRO augmentation therapy.

### 2.3. Outcome indicators

The effectiveness of different shoulder joint drug injection therapies on rotator cuff injuries. And the literature has clear criteria for effectiveness evaluation.

### 2.4. Exclusion criteria

Duplicate publications. Conference papers and letters. Studies with incomplete or incorrect data information and fruitless contact with authors.

### 2.5. Search strategy

Computer searches for relevant RCTs in PubMed, The CochraneLibrary (2015, Issue 10), EMbase, Chinese national knowledge infrastructure, Chinese BioMedical Literature on disc, China Science and Technology Journal Database, Wan-Fang databases. English search terms mainly included “Rotator cuff injury,” “corticosteroid,” “PRP,” “prolotherapy,” “Hyaluronic Acid,” “Hyaluronic acid,” “distension,” “distension.” The search was performed using subject terms paired with free words, using the corresponding Boolean logic operator linkage. No language restrictions were applied, and the search period was from April 19, 2013 to April 19, 2022 (the last decade).

### 2.6. Literature screening and data extraction

Two investigators independently screened the literature according to the inclusion and exclusion criteria, extracted the data according to the pre-defined data extraction form, and cross-checked the data, and in case of disagreement, agreed through mutual discussion or referred to the third investigator for decision. Data extraction included basic information about the literature (literature number, title, first author, year of publication, etc), study-related information (mean age of patients, gender composition, disease classification, diagnostic criteria, interventions, frequency of interventions, duration of treatment, follow-up time, efficacy evaluation criteria, and data on outcome indicators) and relevant elements of risk of bias evaluation.

### 2.7. Statistical analysis

Trials with 4 and more arms were first split into all possible combinations of 2 arms and evidence network plots were drawn for the comparison of each treatment measure.^[9]^ Comparison-corrected funnel plots were produced to evaluate the interventions for small sample effects or publication bias. Inconsistency factors and their 95% confidence interval (CI) were calculated to evaluate the consistency of each closure, with the lower 95% CI equal to 0 considered as good consistency, otherwise the closure was considered to have significant inconsistency.^[10]^ Using a Bayesian Markov Chain Monte Carlo (MCMC) random effects model, 4 chains were used for simulation, and the number of iterations was set to 50,000, with the first 20,000 used for annealing to eliminate the effect of initial values and the second 30,000 used for sampling, to calculate the ratio of effectiveness of each treatment measure compared (odd ratio) values and 95% CI with 95% CI not including 1. *P* < .05 was considered statistically significant.^[11]^ Sensitivity analysis was performed using MCMC fixed-effects model to evaluate the stability of the study results, and the parameters were set as in the random-effects model. SUCRA graphs were plotted to predict the ranking of the efficacy of each treatment measure, with a larger area under the curve (0%–100%) indicating a better treatment measure.^[12]^ The above graphs were plotted using Stata 16.0 for statistical analysis.

## 3. Results

### 3.1. Literature search results

**Figure 1.**
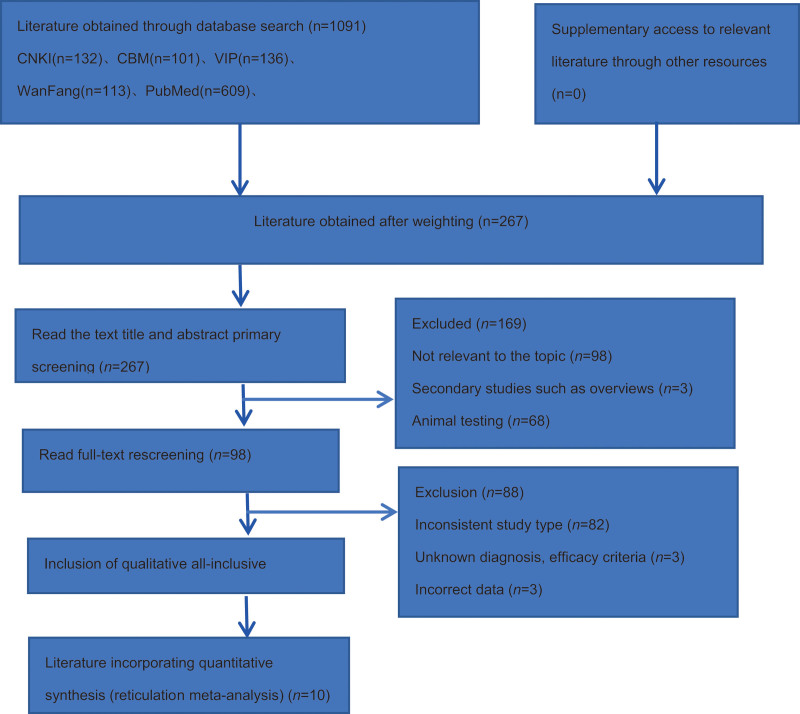
Flow chart of article screening and selection process. Note: CNKI = Chinese national knowledge infrastructure, CBM = Chinese BioMedical literature on disc, VIP = China science and technology journal database.

The databases were initially checked for 1091 papers, and 10 papers were obtained after ranking by NoteExpress 3.6 software, which were finally included in 10 RCTs after initial screening of titles and abstracts and re-screening by reading the full text.^[13–22]^ The flow chart of literature screening is shown in Figure 1.

### 3.2. Basic characteristics of the included literature

A total of 861 patients with clinically confirmed rotator cuff injury in 10 studies, all with a mean age greater than 40 years, reported comparable or non-significant differences in age, sex, duration and severity of disease between groups. 1 study was a 4-arm trial,^[15,18]^ and the others were 2-arm trials. A total of 7 measures in 4 components of combination therapy involving COR, PRP, HA, and PRO therapy. The duration of observation of outcome indicators was mostly 6 weeks. The basic characteristics of the included literature are shown in Table 1.

**Table 1:** General characteristics and quality assessment of the studies included in this network meta-analysis.

Inclusion in the study	Treatment group 1			Treatment group 2			Ending indicators
	Interventions	Number of cases (M/F)	Age (yr)	Interventions	Number of cases (M/F)	Age (yr)	
Shashank Yeshwant Kothari 2017	PRP	62 (34/28)	51.9 ± 10.1	COR	62 (29/31)	52.7 ± 8.6	Vas
Haleh Dadgostar2021	PRP	30 (5/25)	57.33 ± 9.80	COR	28 (6/22)	53.60 ± 7.24	Vas
YU CAI2018	HA	44 (24/20)	38.93 ± 7.35	PRP	45 (22/23)	40.56 ± 7.85	Vas
				PRP + HA	48 (26/22)	39.63 ± 7.65	Vas
Yalan Yan2021	PRP + COR	50 (32/18)	42.92 ± 6.88	PRP	50 (29/21)	39.78 ± 5.44	Vas
Jiachen Zhang2020	PRP	27 (18/9)	46.40 ± 9.87	COR	34 (20/8)	43.97 ± 11.98	Vas
Aylin Sari2019	PRP	30 (–/–)	–	PRO	30 (–/–)	–	Vas
				COR	30 (–/–)		
Ferhat Say2016	PRP	30 (10/20)	49.2 ± 7	COR	30 (12/18)	50.2 ± 2.7	Vas
L.I.F.Penning2012	HA	51 (24/27)	53 ± 12	COR	53 (27/26)	52 ± 9	Vas
Ying Peng2019	PRO	50 (17/33)	46.8 ± 9.5	COR	50 (19/31)	47.9 ± 8.8	Vas
Seung Deuk Byun2012	COR + HA	15 (7/8)	53.8 ± 9.8	COR	15 (6/9)	55.4 ± 10.0	

COA+HA = corticosteroid injection + hyaluronic acid injection, COR = corticosteroid injection therapy, HA = hyaluronic acid injection therapy, PRO = prolotherapy therapy, PRP = platelet-rich plasma injection therapy, PRP+COR = platelet-rich plasma injection+ Corticosteroid injection, PRP+HA = platelet-rich plasma injection + hyaluronic acid injection.

### 3.3. Results of the mesh meta-analysis

#### 3.3.1. Evidence network diagram.

**Figure 2.**
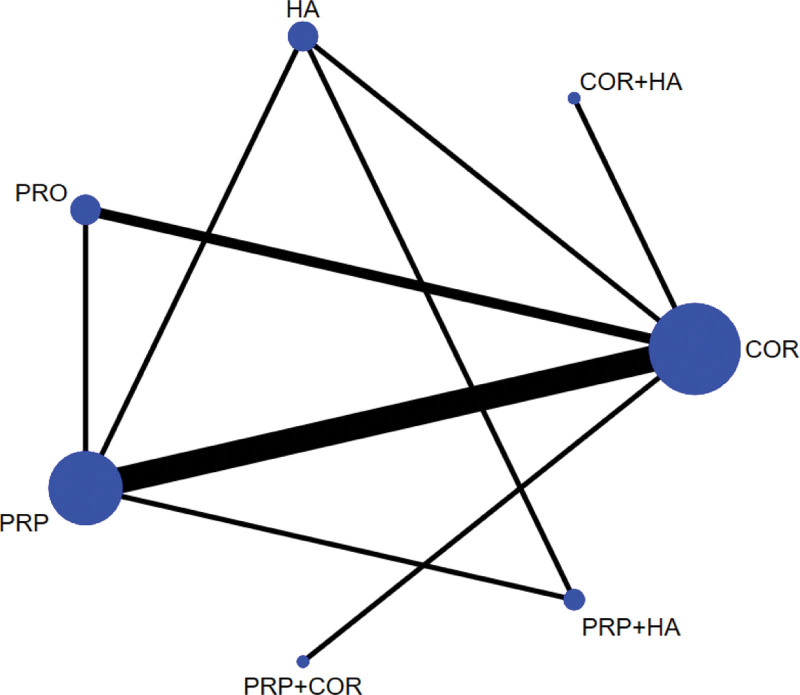
Evidence network for the effectiveness of different joint cavity drug injections for rotator cuff injuries. Note: COR = corticosteroid injection therapy, PRP = platelet-rich plasma injection therapy, HA = hyaluronic acid injection therapy, PRO = prolotherapy therapy, COA + HA = corticosteroid injection + hyaluronic acid injection, PRP + HA = platelet-rich plasma injection + hyaluronic acid injection, PRP + COR = platelet-rich plasma injection + corticosteroid injection. A line between points indicates direct comparative evidence for the 2 interventions; no line indicates no direct comparative evidence.

Seven treatment measures, 20 different two-by-two comparisons can be formed. A total of 8 direct comparisons exist for the 10 included studies, with no direct research evidence for the remaining 14 comparisons, whose efficacy comparisons will be generated by indirect comparisons from a reticulated Meta-analysis. Figure 2 shows the network diagram of the evidence for the 4 treatment measures of the 10 included RCTs. In the figure, there are connecting lines between the dots indicating direct comparative evidence for the 2 interventions, and no connecting lines indicating no direct comparative evidence.

#### 3.3.2. Contribution of the 4 interventions to the results of the reticulated meta-analysis.

**Figure 3.**
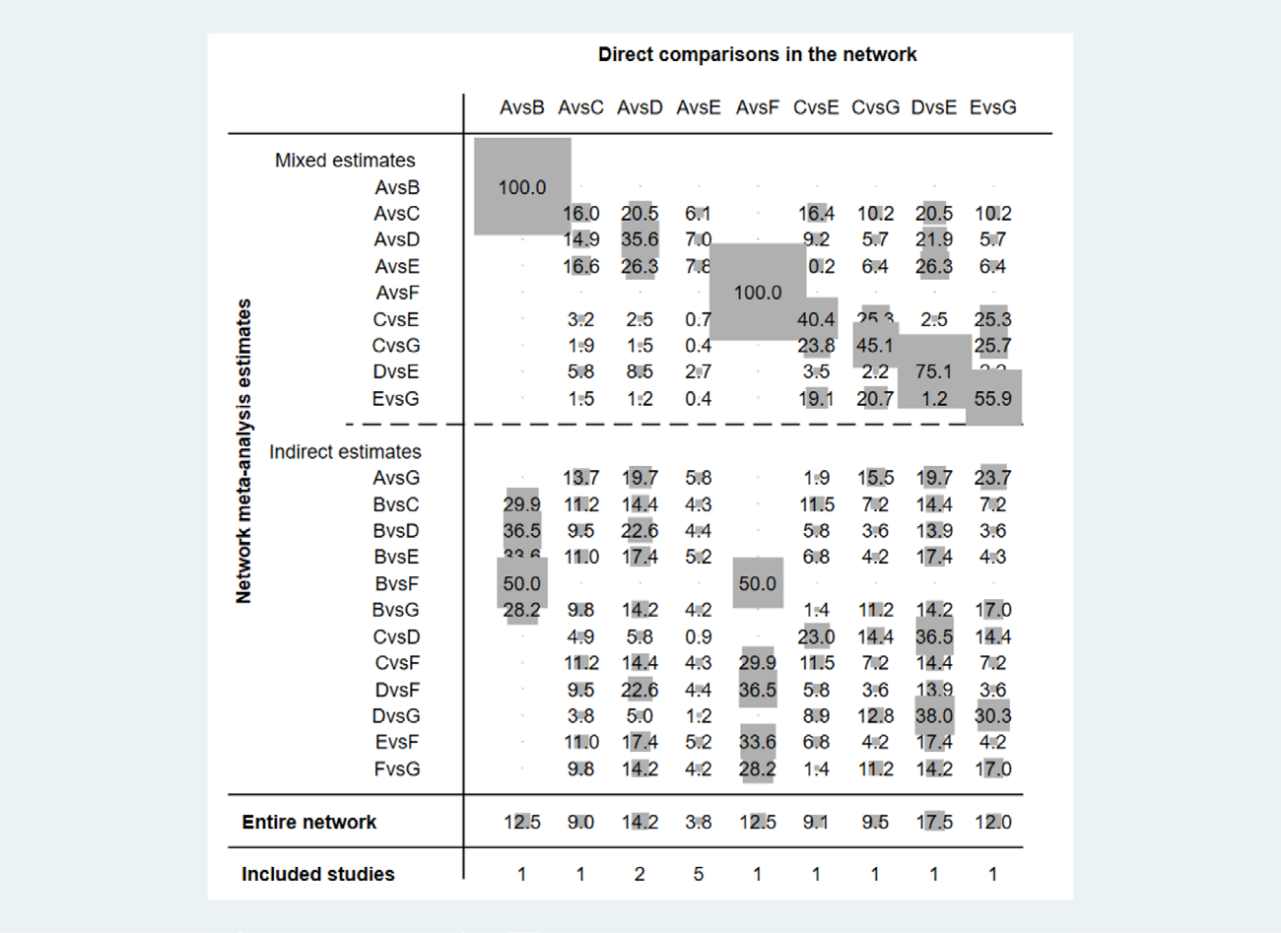
Contribution of the results of the reticulated meta-analysis of different joint cavity drug injection interventions. Note: A = Corticosteroid injection therapy, B = COA + HA: Corticosteroid injection + Hyaluronic acid injection, C = Hyaluronic acid injection, D = Prolotherapy therapy, E = Platelet-rich plasma injection, F = Platelet-rich plasma injection + Corticosteroid injection, G = Platelet-rich plasma injection + Hyaluronic acid injection.

For further analysis of the impact and contribution of each direct comparison to the network meta-analysis, the value of the contribution of each group of direct comparisons to the study is indicated by gray circles and weight scores, Figure 3 shows the impact of different direct comparisons on the results of the mesh meta-analysis and the results of the whole network mesh meta-analysis in this study, and its results suggest that for the whole network meta-analysis, the direct comparison of PRO versus the direct comparison of platelet-rich plasma injection control had the highest contribution (17.5%), followed by the direct comparison of corticosteroid injection and PRO (14.2%) for the whole network meta-analysis.

#### 3.3.3. Inconsistency test.

**Figure 4.**
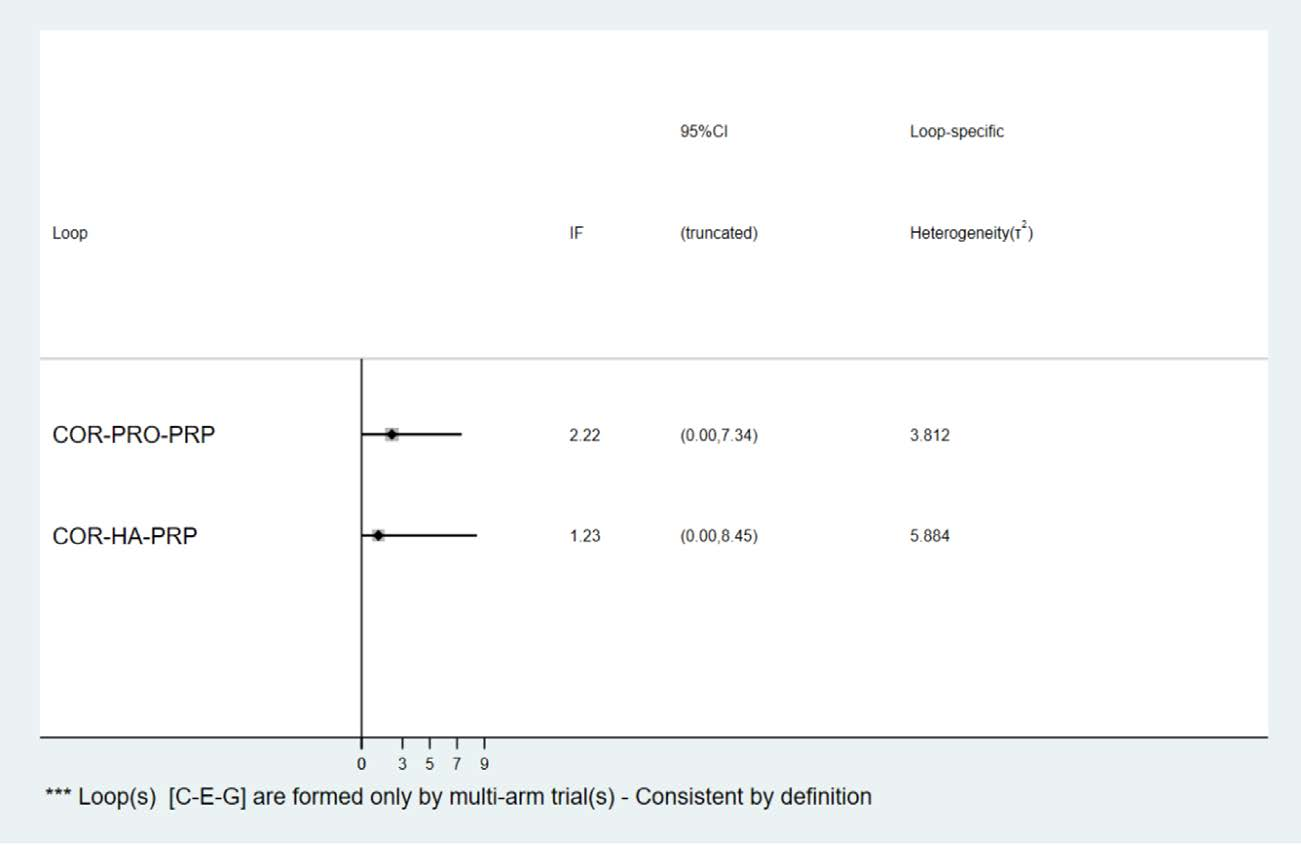
Results of inconsistency test for total efficiency. Note: COR = Corticosteroid injection therapy, PRP = Platelet-rich plasma injection therapy, HA = Hyaluronic acid injection therapy, PRO = Prolotherapy therapy, COA + HA = Corticosteroid injection + Hyaluronic acid injection, PRP + HA = Platelet-rich plasma injection + Hyaluronic acid injection, PRP + COR = Platelet-rich plasma injection + Corticosteroid injection.

**Figure 5.**
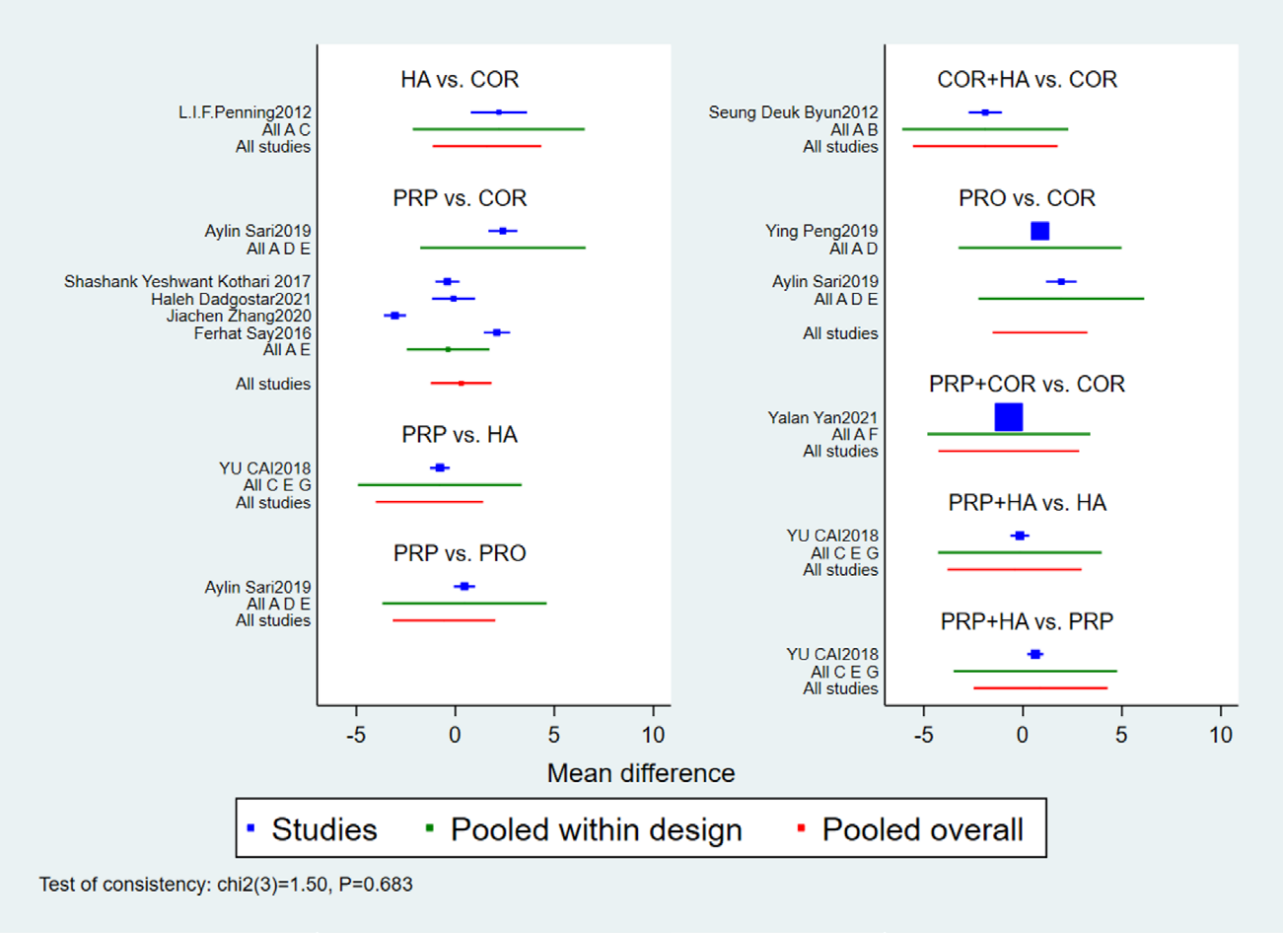
The results of the test of inconsistency between direct comparison and indirect comparison of total efficiency. Note: COR = Corticosteroid injection therapy, PRP = Platelet-rich plasma injection therapy, HA = Hyaluronic acid injection therapy, PRO = Prolotherapy therapy, COA + HA = Corticosteroid injection + Hyaluronic acid injection, PRP + HA = Platelet-rich plasma injection + Hyaluronic acid injection, PRP + COR = Platelet-rich plasma injection + Corticosteroid injection.

The global inconsistency test suggested *P* = .683 > .05, indicating a significant consistency model, as detailed in Figure 4; 2 trilateral rings (corticosteroid injection-PRO-PRP, HA-PRP-PRP + HA). The consistency of the findings of each closed-loop study was tested, and the results showed inconsistency factors bound 1.42 to 2.22, and the lower limit of 95% CI were 0.00, as detailed in Figure 5, indicating good consistency of each closed-loop.

#### 3.3.4. Small sample effect detection.

**Figure 6.**
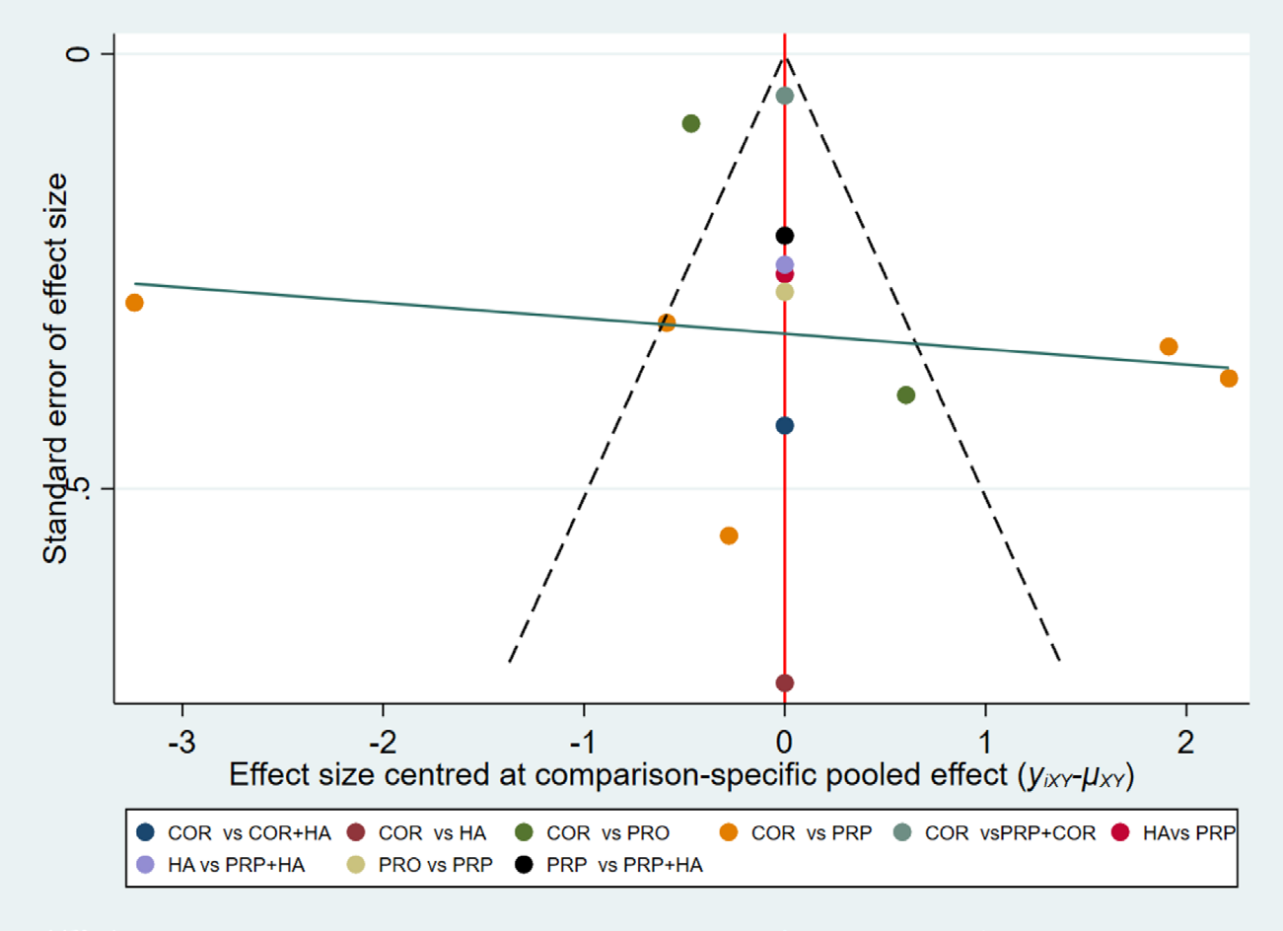
Comparison of total efficiency of different articular cavity drug injections for rotator cuff injuries-corrected funnel plot. Note: COR = Corticosteroid injection therapy, PRP = Platelet-rich plasma injection therapy, HA = Hyaluronic acid injection therapy, PRO = Prolotherapy therapy, COA + HA = Corticosteroid injection + Hyaluronic acid injection, PRP + HA = Platelet-rich plasma injection + Hyaluronic acid injection, PRP + COR = Platelet-rich plasma injection + Corticosteroid injection.

A comparison-corrected funnel plot was made for the 4 interventions in the 10 papers included in this study, where different colored points in the funnel plot indicated different direct two-by-two comparisons and the number of the same colored points indicated the number of that two-by-two comparison in the original study. If the funnel plot is symmetrical, it indicates that there is no significant small sample effect or publication bias. From Figure 6, it can be seen that the funnel plot is basically symmetrical, indicating that there is little possibility of small sample effect or publication bias in the study.

#### 3.3.5. Results of reticulated meta-analysis.

The results of the random-effects model mesh Meta-analysis based on the Bayesian theory MCMC method showed (Table 2) that the results of the comparison of 4 drug injections and combination modalities revealed that the efficacy of corticosteroid injection + hyaluronic acid injection was found to be better than platelet-rich plasma injection + corticosteroid injection and platelet-rich plasma injection + hyaluronic acid injection among the combination treatment methods, and the results of the comparison with single treatment revealed that the efficacy of corticosteroid injection + hyaluronic acid injection was better than hyaluronic acid injection, corticosteroid injection, platelet-rich plasma injection and PRO treatment measures.

**Table 2:** Results of the reticulated meta-analysis of the effectiveness of different articular cavity drug injections in the treatment of rotator cuff injuries.

3.50 (−1.07, 8.07)	3.08 (−2.02, 8.19)	2.77 (−1.60, 7.13)	2.19 (−1.77, 6.15)	1.90 (−1.75, 5.55)	1.19 (−3.91, 6.29)	**COR + HA**
2.31 (−2.18, 6.80)	1.89 (−3.14, 6.93)	1.58 (−2.71, 5.86)	1.00 (−2.87, 4.88)	0.71 (−2.85, 4.27)	**PRP + COR**	−1.19 (−6.29, 3.91)
1.60 (−1.14, 4.34)	1.18 (−2.38, 4.75)	0.87 (−1.53, 3.26)	0.29 (−1.24, 1.82)	**COR**	−0.71 (−4.27, 2.85)	−1.90 (−5.55, 1.75)
1.31 (−1.41, 4.02)	0.89 (−2.49, 4.27)	0.57 (−2.02, 3.16)	**PRP**	−0.29 (−1.82, 1.24)	−1.00 (−4.88, 2.87)	−2.19 (−6.15, 1.77)
0.73 (−2.80, 4.27)	0.32 (−3.85, 4.49)	**PRO**	−0.57 (−3.16, 2.02)	−0.87 (−3.26, 1.53)	−1.58 (−5.86, 2.71)	−2.77 (−7.13, 1.60)
0.42 (−2.97, 3.80)	**PRP + HA**	−0.32 (−4.49, 3.85)	−0.89 (−4.27, 2.49)	−1.18 (−4.75, 2.38)	−1.89 (−6.93, 3.14)	−3.08 (−8.19, 2.02)
**HA**	−0.42 (−3.80, 2.97)	−0.73 (−4.27, 2.80)	−1.31 (−4.02, 1.41)	−1.60 (−4.34, 1.14)	−2.31 (−6.80, 2.18)	−3.50 (−8.07, 1.07)

COA+HA = corticosteroid injection + hyaluronic acid injection, COR = corticosteroid injection therapy, HA = hyaluronic acid injection therapy, PRO = prolotherapy therapy, PRP = platelet-rich plasma injection therapy, PRP+COR = platelet-rich plasma injection + corticosteroid injection, PRP+HA = platelet-rich plasma injection + hyaluronic acid injection.

#### 3.3.6. Efficacy ranking of treatment measures.

**Figure 7.**
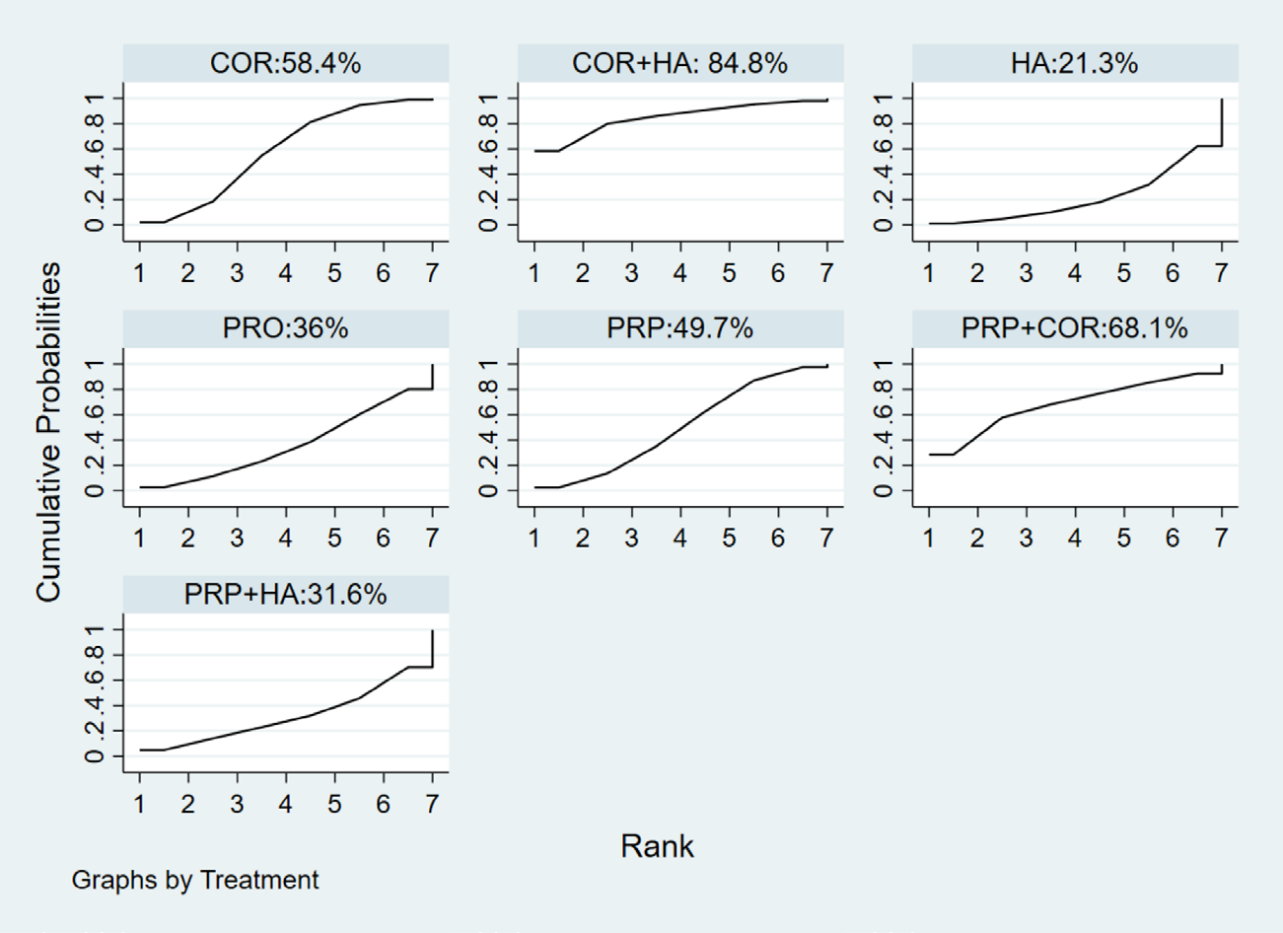
Efficacy ranking of different articular cavity drug injections for rotator cuff injury SUCRA. Note: COR = Corticosteroid injection therapy, PRP = Platelet-rich plasma injection therapy, HA = Hyaluronic acid injection therapy, PRO = Prolotherapy therapy, COA + HA = Corticosteroid injection + Hyaluronic acid injection, PRP + HA = Platelet-rich plasma injection + Hyaluronic acid injection, PRP + COR = Platelet-rich plasma injection + Corticosteroid injection.

The SUCRA curve was plotted according to the results of the MCMC method random effects model comparison, and the area under the curve was used to predict the ranking of efficacy, see Figure 7. The area under the curve of corticosteroid injection + hyaluronic acid injection was 84.8%, and the results of the 4 treatment measures were as follows: corticosteroid injection + hyaluronic acid injection > platelet-rich plasma injection + corticosteroid injection > corticosteroid injection > platelet-rich plasma injection > PRO > platelet-rich plasma injection + hyaluronic acid injection > hyaluronic acid injection. The ranking also showed that corticosteroid injection + hyaluronic acid injection was the most effective.

## 4. Discussion

Rotator cuff injury is a common musculoskeletal disorder that causes shoulder pain and impaired shoulder movement due to injury to rotator cuff related muscles and tendons.^[23]^ The current treatment for rotator cuff loss injuries is divided into surgical and non-surgical conservative treatments. The American Academy of Orthopedic Surgeons, in its 2013 proposed criteria for the use of optimal management of total rotator cuff tears, states that the recommended non-surgical treatment of rotator cuff injuries consists of 5 main areas: pharmacotherapy and corticosteroid injections (SAI), information about symptom control, activity level modification education about symptom control, activity level adjustment, home training and prognosis, manipulative therapy, functional training and physiotherapy.^[24]^ Among them, injection therapy is one of the commonly used conservative treatments.^[25]^ Current injection therapies for rotator cuff injuries include COR, PRP, HA, and PRO.

Intra-articular cavity injection of steroids has the effect of reducing the synovial inflammatory response and effectively relieving aseptic inflammation of the shoulder capsule and surrounding tissues. Dimitrios Georgianos et al^[26]^ argued that physical therapy and active and passive exercise, combined with oral medication and/or intra-articular corticosteroid injections, are considered to be the most important in the conservative management of ACS. The recommendation index for steroid drug injection therapy in the 2022 clinical guidelines for the management of periarthritis of the shoulder in Chinese orthopedics is B.^[27]^

Platelet-rich plasma has a regenerative repair effect and is characterized by using the patient’s own blood, collecting large amounts of platelet-rich plasma prior to injection, and injecting it into the area of bone or soft tissue injury, which can effectively help revascularize the injured area and promote tissue healing. It has been shown that platelet-derived growth factors and vascular permeability factors contained in platelet-rich plasma can promote local production of interleukin factors, induce fibroblast differentiation, and inhibit further development of inflammation during the early stages of recovery from rotator cuff injury, thereby relieving patient pain.^[28]^ Yan Yalan^[16]^ evaluated the clinical effect of platelet-rich plasma combined with drug injection for rotator cuff injury by selecting 100 patients with rotator cuff injury.

Proliferative injection therapy (prolotherapy) is a recently applied treatment for diseases of the musculoskeletal system, which is based on the principle of injecting a highly concentrated glucose injection of 12.5% to 25% into a painful ligament or tendon that can promote tissue regeneration and initiate the body’s self-repair system, by stimulating a transient inflammatory response in order to achieve repair and regeneration.^[29,30]^ Seven et al^[31]^ found that patients with rotator cuff injuries showed more significant improvements in VAS scores, SPADI index, and western ontario rotatory cuff index within 1 year of receiving augmentation injections compared to patients who did not receive injections.

Sodium hyaluronate is an intra-articular lubricant that not only lubricates the joint but also covers and protects the articular cartilage, inhibits the inflammatory response, and prevents degenerative changes in the articular cartilage.^[32]^ Yozo Shibata et al^[33]^ concluded that sodium vitrate is an injection that can be as effective as steroids in the treatment of rotator cuff tears. They compared the test group (sodium vitreous acid injection) and the control group (steroid injection) separately for total rotator cuff tear patients at 4 and 24 weeks post-injection for treatment efficacy and satisfaction.

A variety of shoulder joint drug injections have been widely used in the clinical treatment of rotator cuff injuries, and their efficacy has been recognized by most patients and physicians. However, there has been controversy about the different drugs and their clinical effectiveness, and there is no complete agreement on the choice of different injectables. The existing original studies are mostly comparisons between single drug injections and lack direct comparisons between classes of multiple drug injections, which affects the comprehensiveness of the efficacy evaluation of different shoulder drug injections. Therefore, more reliable evidence-based medical evidence for a more comprehensive understanding and objective evaluation of the modality of drug injections in the rotator cuff joint is necessary to conduct more comprehensive clinical studies to provide more reliable evidence for selecting the best drug injection therapy for rotator cuff injuries. This research study included 10 RCTs comparing steroid injection therapy, PRP, high glucose augmentation therapy, and sodium glutamate injection therapy available in the database, with a combined sample size of 861 cases, and included 7 therapeutic measures. Using reticulated META for analysis, the results revealed the best non-surgical efficacy of steroid combined with sodium glacial injection for the treatment of rotator cuff injuries among the 7 treatment measures included. Also study comparison-corrected funnel plots did not reveal significant asymmetry, suggesting no significant small sample effect or publication bias, inconsistency tests suggested good consistency across closed loops, and the random-effects model efficacy ranking in sensitivity analysis was the same as the fixed-effects model, indicating stable findings for the 7 treatment measures. The SUCRA plot also showed that corticosteroid injection + hyaluronic acid injection was the first with 84.8% area under the curve. 7 treatment measures were ranked as follows: corticosteroid injection + hyaluronic acid injection > platelet-rich plasma injection + corticosteroid injection > corticosteroid injection > platelet-rich plasma injection > PRO > platelet-rich plasma injection + hyaluronic acid injection > hyaluronic acid injection. The ranking also showed that corticosteroid injection + hyaluronic acid injection was the most effective.

This study compared the efficacy of different rotator cuff injury drug injection therapies through reticulated Meta-analysis, solving the problem of lack of direct comparison of different rotator cuff injury injection methods in the original study, and providing a more comprehensive and systematic understanding of the effectiveness of different drug injections in the treatment of rotator cuff injury therapies. More reliable evidence-based medical evidence is available for the selection of the best treatment option for rotator cuff injuries. The results of this study suggest that corticosteroid injections combined with hyaluronic acid therapy is the best non-surgical injection therapy for rotator cuff injuries. Based on this study, we recommend that corticosteroid injections combined with hyaluronic acid injections can be used in the conservative non-surgical clinical management of rotator cuff injuries.

Based on the shortcomings of existing studies, the small amount of relevant literature, and the low quality of included studies and small sample size in this study, the findings still need to be validated by a large number of well-designed, methodologically appropriate, high-quality, large-sample RCTs.

## 5. Limitations

This study attempted to gather as much clinical information as possible on the different shoulder drug injection therapies used to treat rotator cuff injuries clinically, but there are still some limitations to our study. Based on the shortcomings of the existing studies, the small amount of relevant literature, and the small sample size included in this study, the findings still need to be validated by a large number of well-designed, methodologically sound, high-quality, large sample RCTs.

## Author contributions

**Conceptualization:** Feiyan Cai, Wei Zhang.

**Data curation:** Fang Zhi,Xingzhen Lin.

**Formal analysis:** Liming Xiong, Xingzhen Lin.

**Investigation:** Jinglin Hu.

**Resources:** Jinglin Hu, Fang Zhi.

**Software:** Xingzhen Lin.

**Supervision:** Fang Zhi, Feiyan Cai.

**Writing – original draft:** Xingzhen Lin, Fang Zhi.

**Writing – review & editing:** Xingzhen Lin.
